# Landscape management can foster pollinator richness in fragmented high-value habitats

**DOI:** 10.1098/rspb.2024.2686

**Published:** 2025-02-05

**Authors:** Carolin Biegerl, Andrea Holzschuh, Benjamin Tanner, Douglas Sponsler, Jochen Krauss, Jie Zhang, Ingolf Steffan-Dewenter

**Affiliations:** ^1^ Department of Animal Ecology and Tropical Biology, Biocenter, University Würzburg, Am Hubland, Würzburg 97074, Germany

**Keywords:** wild pollinators, calcareous grassland, habitat area, agri-environmental schemes, landscape management, nature conservation

## Abstract

Pollinator diversity is declining due to habitat loss, low habitat quality, limited habitat connectivity and intensification of agriculture in remaining high-value habitats within human-dominated landscapes, such as calcareous grasslands. Options to increase the local area of protected habitats are often limited. Therefore, we asked how local habitat quality as well as agri-environmental schemes (AES) and configuration of the surrounding landscape can contribute to the preservation of pollinator diversity. We sampled bees, butterflies and hoverflies in 40 calcareous grasslands in Germany, and assessed the effects of calcareous grassland area, quality and connectivity, agricultural configuration, and AES on species richness and abundance. While calcareous grassland area was an important predictor for bee and butterfly species richness, with strongest effects sizes for endangered species, local flower resources and nesting sites and landscape characteristics such as small field size, high proportion of organic fields and connectivity with other grasslands significantly enhanced pollinator richness with responses differing among the three studied taxa. In contrast to expectations, AES flowering fields did not benefit pollinator communities in grasslands. We conclude that improving local habitat quality in combination with targeted landscape management are effective measures to promote pollinator richness in highly fragmented protected grassland.

## 1. Introduction

Habitat loss and land-use change have been the main drivers of the rapid decline of insects in recent decades [1–3]. The loss of wild pollinating insects in particular poses a serious threat to humanity and nature, as 35% of crops [4] and about 88% of all angiosperms worldwide benefit from pollination by insects or other animals [5]. In Central Europe, semi-natural habitats like calcareous grasslands are major remaining refuges for wild pollinators in human-dominated agricultural landscapes. Crop fields alone are unlikely to fulfil the actual needs of pollinators, whereas calcareous grasslands are valuable habitats because of their ability to provide a variety of floral resources and nesting sites [6,7]. The low disturbance intensity of these grasslands allows many pollinators, including many endangered species, to survive and provide pollination services to adjacent crop fields [8,9]. Calcareous grasslands were once an integral part of the landscape and were created by extensive grazing. However, due to the unprofitability of extensive farming and the continued agricultural intensification, calcareous grasslands have become increasingly fragmented and isolated [10].

Through the lens of island biogeographic [11] and ecosystem decay theory [12], the decreasing area of calcareous grasslands should be accompanied by a loss of within-patch biodiversity and habitat quality, including floral and nesting resources. The positive effect of habitat area [13,14] and flower resources on pollinators has been shown frequently [15,16]. However, nesting resources are also important for wild bees, but this driver has rarely been studied, and even more rarely in combination with calcareous grassland areas and floral resources in the context of grasslands [17,18]. Above the scale of a single patch, the proportion of calcareous grasslands in the landscape is expected to affect pollinator diversity by determining habitat connectivity [17,19]. The positive effects of a higher cover and connectivity of calcareous grasslands could be due to more abundant, diverse and temporally continuous floral resources. Additionally, the higher probability of dispersal events in a well-connected metacommunity decreases the risk of populations becoming extinct in fragmented habitats [20].

To reach other calcareous grassland fragments, pollinators often have to cross the surrounding agricultural matrix. However, it is unclear how hostile this matrix is to pollinators found in protected grasslands [21,22]. In addition, it is difficult in practice to foster pollinators by increasing calcareous grassland and improving habitat connectivity, as land is a valuable commodity, and much land is already under cultivation. Therefore, a comparison between the classically considered variables of calcareous grassland area and connectivity and the changes that can be achieved through land management is desirable and has not been widely studied. Pollinators often use flower resources in agricultural fields and adjacent perennial habitats [23,24], but it has rarely been studied how the composition and configuration of the agricultural matrix contribute to pollinator diversity in grassland patches. Smaller crop fields in the surrounding landscape, for instance, can lead to higher structural and vegetative diversity, as more crop edges lead to the presence of uncultivated field margins and hedgerows [25]. The lower management intensity of field edges leads to reduced use of fertilizers and insecticides, which can support higher levels of flower density and pollinator colonization in field edges compared with centres [26]. This together could result in more area that provides food and nesting resources [27,28]. Another approach to enhancing agricultural land for biodiversity is through agri-environmental schemes (AES), such as organic farming and flowering fields. Both AES are expected to benefit pollinators on calcareous grasslands because the absence of pesticides in organically managed fields and the establishment of flowering fields lead to a higher floral cover [29,30] and improve connectivity at the landscape scale [31,32]. However, there are only a few studies focusing on landscape-scale effects of organic farming [30,33] and knowledge about the effect of AES on pollinators in high-value habitats is lacking.

This study assesses the effects of calcareous grassland area and quality, habitat connectivity, agricultural landscape configuration and AES, on wild bee, butterfly and hoverfly species richness and abundance in calcareous grasslands. Consistent with the island biogeographic and ecosystem decay theory, we expect that (i) pollinator species richness and abundance will increase with calcareous grassland area and connectivity, as well as with patch-scale habitat quality. With respect to the agricultural matrix, (ii) smaller field sizes in the surrounding landscape should lead to higher pollinator species richness and abundance on calcareous grasslands through more small-structured crop edges. In addition, the presence of AES such as organic farming and flowering fields should increase pollinator species richness and abundance by augmenting food resources on the landscape scale.

## 2. Methods

### (a) Study region and sampling sites

**Figure 1.**
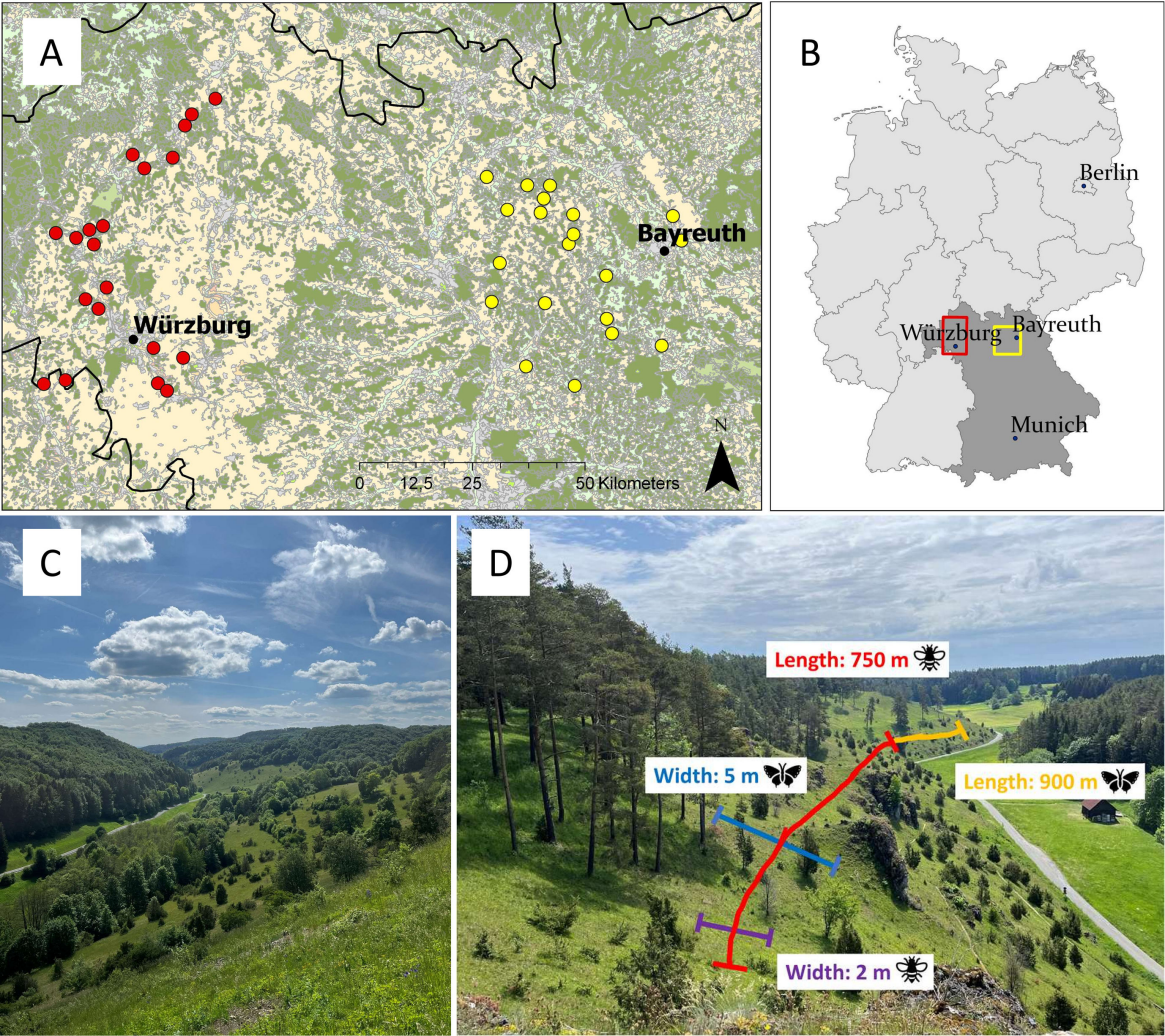
(A,B) Locations of the 40 study sites (calcareous grasslands) in two study regions: Lower (red) and Upper Franconia (yellow). Map source: Corine Landcover 2018 © GeoBasis-DE / BKG (2023). (C) Calcareous grassland in Upper Franconia. (D) Visualization of variable transect walks for bees, hoverflies and butterflies on calcareous grassland in Upper Franconia.

The study was conducted in 2022 across two study regions in northern Bavaria, Germany: Lower Franconia and Upper Franconia (figure 1). The two regions differ in their annual mean temperature of 2022 (11.06°C in Lower and 10.29°C in Upper Franconia), annual precipitation 2022 (565.6 mm in Lower and 698.8 mm in Upper Franconia) and altitude (284 m above sea level in Lower and 465 m above sea level in Upper Franconia) [34–36]. The geology of Lower Franconia is characterized by shell limestone, whereas Jurassic limestone is dominant in Upper Franconia [37]. In both regions, calcareous grasslands were formerly widespread on slopes of the valleys [38], and are nowadays embedded in an agricultural matrix characterized by annual crop fields and, especially in Lower Franconia, vineyards.

Forty calcareous grasslands (20 grasslands per study region) were selected as study sites to test the effects of local and landscape variables on wild pollinator species richness and abundance, and flower resources. To cover a broad gradient of local and landscape variables, study sites were selected with different habitat areas (ranging from 0.07 to 31.54 ha), and different habitat connectivity, measured as the proportion of calcareous grasslands in a buffer with a 2 km radius around the study sites (ranging from 0% to 4.7%) excluding the study site area. Distance between study sites was always at least 2 km to ensure independent landscapes and species communities.

### (b) Wild pollinators

Bees (Hymenoptera: Apiformes), hoverflies (Diptera: Syrphidae), butterflies (Lepidoptera: Hesperioidea and Papilionoidea) and burnet moths (Lepidoptera: Zygaenidae) were sampled on the study sites five times from April to August 2022. In the following, butterflies always include burnet moths. We analysed bumblebees separately from other wild bees, as bumblebees differ from other bees in their ecology and sociality, and we omitted honeybees altogether. In the following, the term solitary bees refers to all bees except bumblebees and honey bees and includes solitary bees as well as primitively eusocial halictid bees. The order of study sites visited was randomized for each sampling round. In order to sample bees, hoverflies and butterflies, variable transect walks with no fixed direction were carried out (figure 1). Transects were not a straight line but were directed in parts of the study site representative and attractive for wild pollinators (i.e. flower patches and bare ground slopes). Transect location could change from round to round and between bee/hoverfly and butterfly sampling. The transect length for bees and hoverflies was 750 m and the transect width was 2 m resulting in a 1500 m² transect area regardless of the study site area. The transect length for butterflies was 900 m and the transect width was 5 m resulting in a 4500 m² transect area to account for higher mobility of butterflies. Transect time was 45 min. Sampling was conducted from 9:00 to 17:00 during suitable weather conditions (temperatures above 13°C in the sun, low wind (<3 bft) and no rain). Each study site was sampled at least once in the morning, noon and afternoon and changed from round to round. One single person carried out collections and taxonomic identification in the field for bees and hoverflies and another person for butterflies. Wild pollinators were caught with a net if not identified on the wing. Wild pollinators which could not be identified to species level in the field were collected and identified in the laboratory; otherwise, the pollinator was released after identification. Bumblebee queens were not collected and were identified to species level in the field. Bee and butterfly species were classified as endangered or not endangered according to the Red List of Bavaria [39]. Too few endangered hoverfly and bumblebee species were sampled to warrant a separate analysis of these taxa. Pollinator species richness and abundance were pooled across rounds. Due to the equal sampling size in time and area on all study sites, pollinator species richness is equivalent to species density per transect and pollinator abundance is equivalent to population density per transect, rather than the total species number or total abundance of the calcareous grassland fragment. Following frequently used terminology including recent studies [12,40,41], we use the terms species richness and abundance throughout the manuscript.

### (c) Local and landscape variables

To estimate the influence of local variables, we used calcareous grassland area and habitat quality, consisting of the variables flower richness, flower cover and nesting sites on the transects. The calcareous grassland area is the area of the sampled calcareous grassland fragment and was calculated in Esri ArcGIS Pro using satellite and aerial imagery from the layer ‘World Imagery’ [42]. As a measure of flower richness, each flowering plant species was recorded during the transect walk for each sampling round and bee/hoverfly and butterfly transect separately. Flower cover on the transect was estimated for all flowering vascular plants in cm² and then converted to per cent cover. Nesting sites is the estimated percentage of the cover of potential nesting sites for solitary bees on the transect. Potential nesting sites for ground-nesting solitary bees are slopes with open ground and loamy sandy spots [43,44], whereas above-ground-nesting bees rely on dead wood and hollow stems [45]. Suitable open soil spots and the surface of dead wood were estimated in m² and then converted to percentage cover. Flower resources (but not nesting resources) were correlated with calcareous grassland area. Flower richness, flower cover and nesting sites were averaged for each plot across rounds.

Landscape variables were calculated within a 2 km radius around the study site centre, to estimate the influence of landscape composition and configuration. The landscape analysis was conducted within the 2 km radius because it represents a reasonable pollinator foraging and dispersal range [19,46–48]. To analyse the influence of habitat connectivity on wild pollinator species richness and abundance, the total amount of calcareous grasslands in the surrounding landscape excluding the study site area itself was calculated. To test the influence of the surrounding landscape configuration on wild pollinators in calcareous grasslands, percentage cover of annual crop fields (excluding leys) and mean field size of annual crop fields in hectares were calculated, as a measure of intensity and diversity of the agricultural matrix. As small fields have a higher proportion of edges than large fields and lower management intensity in field edges compared with field centres, smaller fields are expected to lead to more food and nesting resources for pollinators at the landscape scale. In order to understand if and how agri-environmental schemes promote wild pollinators in protected grasslands, the two best-known and most well-established AES were quantified: the percentage cover of organic farming and flowering fields in relation to the total annual crop cover. Organic farming uses fewer pesticides, provides more non-crop vegetation and a more diverse crop rotation than conventional farming [49]. Flowering fields are likewise financially subsidized measures to increase the food resources and shelter for wild plants and animals in arable farming. Both measures are expected to promote wild pollinators even in calcareous grasslands because they lead to a higher flower cover at the landscape scale [29,30]. The calculation of landscape variables was done in ArcGIS Pro 2.2.0 [50]. Aerial photos [42] and data from the biotope mapping Bavaria (Bayerisches Landesamt für Umwelt 2021, https://www.lfu.bayern.de/index.htm) and the integrated management and control system for Bavarian agriculture (InVeKos, Bayerische Landesanstalt für Landwirtschaft 2022) showing detailed information about land-use classes, field crops and agri-environmental schemes (AES) from 2022 were used.

### (d) Statistical analysis

**Figure 2.**
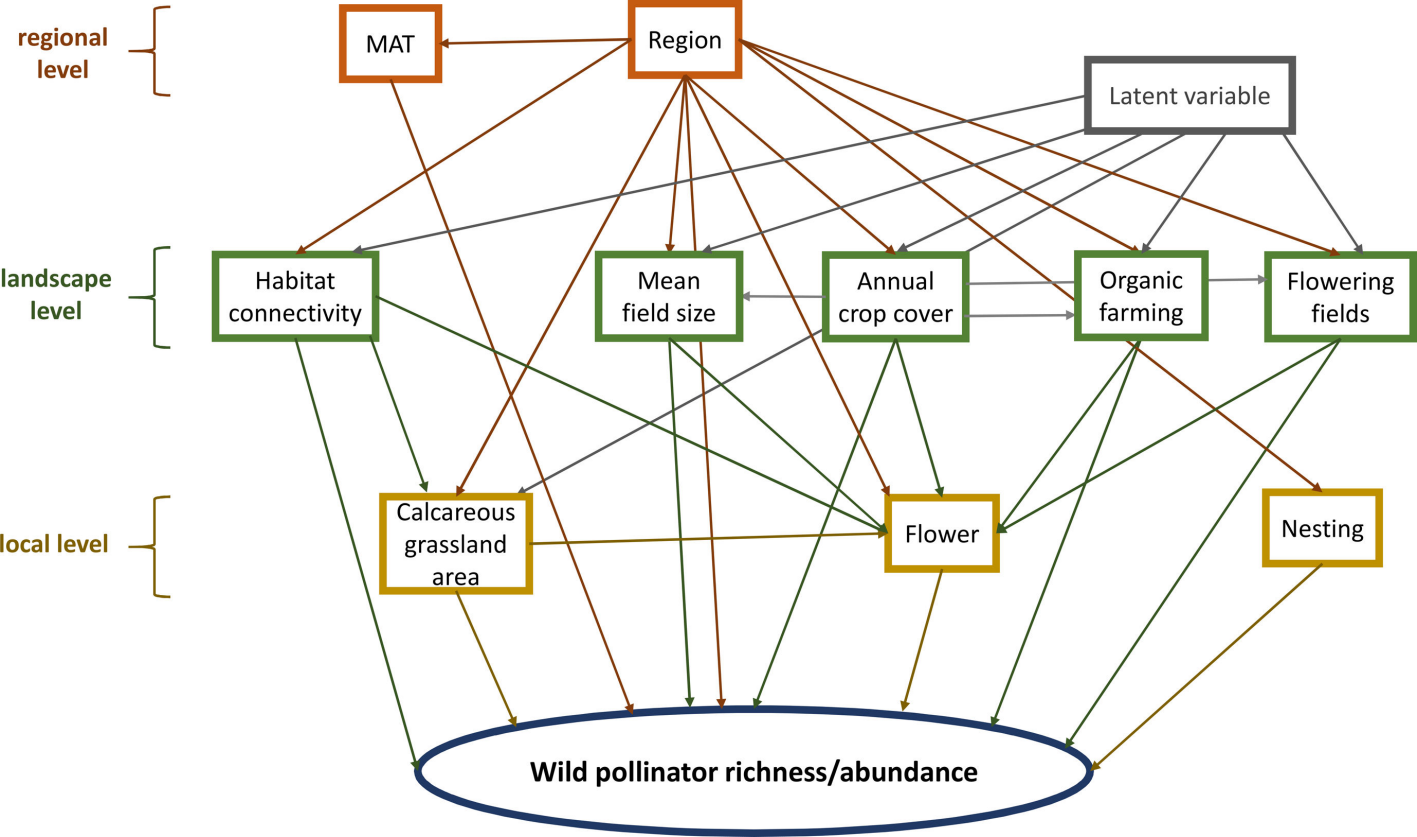
DAG showing the causal structure (arrows) among local, landscape and regional variables (rectangles) hypothesized to be driving wild pollinator species richness and abundance (oval). MAT = mean annual temperature.

We used structural causal modelling to design statistical models to estimate the causal effects of local and landscape variables on pollinator species richness and abundance. Briefly, we constructed a directed acyclic graph (DAG) expressing plausible causal relations among our variables (figure 2), then identified via the back door criterion sets of covariates needed to obtain an unbiased estimate of the causal effect of each explanatory variable [51]. To ensure the appropriateness of the DAG, we tested the DAG-data consistency. We show that our DAG contains no open biasing paths, and all implied independencies are consistent with the observational dataset. We also tested the variables in our DAG for residual spatial autocorrelation using the DHARMa Morans I test for distance-based autocorrelation and multicollinearity using the Variance Inflation Factor (VIF). Despite the advantages of DAGs, possible limitations of the approach are that DAGs build on assumptions based on domain knowledge, literature and the experience of researchers to explain the studied system. The DAG can therefore help to understand complex ecological processes, but there is no guarantee that all assumptions are correct [51]. More details about DAG construction, validation and possible limitations can be found in the supplementary material (electronic supplementary material, methods S1).

The effects of local and landscape variables on pollinator species richness and abundance and flower resources were estimated using generalized linear models (GLM). Response variables were pollinator species richness and abundance, with separate models fitted for each pollinator group and, in the case of bees and butterflies, for endangered species. Local explanatory variables were flower richness and flower cover for bees/hoverflies and butterflies, respectively, nesting sites for solitary bees and calcareous grassland area. Grassland area was log10-transformed to increase linearity. Explanatory landscape variables were habitat connectivity, mean field size, cover of organic farming, cover of flowering fields and annual crop cover. At the regional level, we included mean annual temperature (1970–2010) as well as the region identity (Upper versus Lower Franconia). Models were fitted using a Poisson distribution for pollinator species richness and a negative binomial distribution (to correct over-dispersion) for pollinator abundance. Obtaining unbiased causal estimates for each variable of interest required fitting separate models for local- and landscape-level inferences, since the effects of landscape are partially mediated by local conditions. We also included models in which flower cover and richness were response variables explained by the other explanatory variables. The generalized linear model to analyse the effect of calcareous grassland area and landscape variables was: *x* ⁓ calcareous grassland area + habitat connectivity + mean field size + organic farming + flowering fields + annual crop cover + mean annual temperature + region. The generalized linear model to analyse the effect of habitat quality was: *x* ⁓ calcareous grassland area + habitat connectivity + mean field size + organic farming + flowering fields + annual crop cover + flower richness + flower cover + nesting sites + mean annual temperature + region. The variables included in the models were tested for residual spatial autocorrelation and multicollinearity and no correlations (*p* > 0.05) were identified.

The statistical analyses were performed using the software R v 4.3.1 and RStudio v 2023.06.1 [52]. The package *tidyverse* [53] was used for data handling. GLMs with negative binomial distribution were performed using the package *MASS* [54]. The packages *dagitty* [55], *DHARMA* [56], *car* [57], *ncf* [58] and *performance* [59] were used for model validation. The packages *marginaleffects* [60] and *modelsummary* [61] were used to derive marginal effects of the GLMs. All graphs were generated using R packages *ggplot2* [62] and *ggeffects* [63]. A complete description and reproducible workflow for model fitting, validation and visualization is provided in the electronic supplementary material S1.

## 3. Results

In total, we recorded 231 wild bee species and 10 859 wild bee individuals (of which 21 were bumblebee species and 2830 bumblebee individuals), 90 butterfly species (24 917 butterfly individuals), 62 hoverfly species (1524 hover fly individuals) and 274 flowering plant species on the 40 calcareous grasslands (see electronic supplementary material, figure S3 for species richness and abundance data among sites). We detected 44% of all wild bees known from Bavaria, 48% of butterflies and 16% of hoverflies. Furthermore, 23% of the sampled wild bee species, 33% of butterfly species and 3% of hoverfly species were endangered according to the Red List of Bavaria.

### (a) Local effects

**Figure 3.**
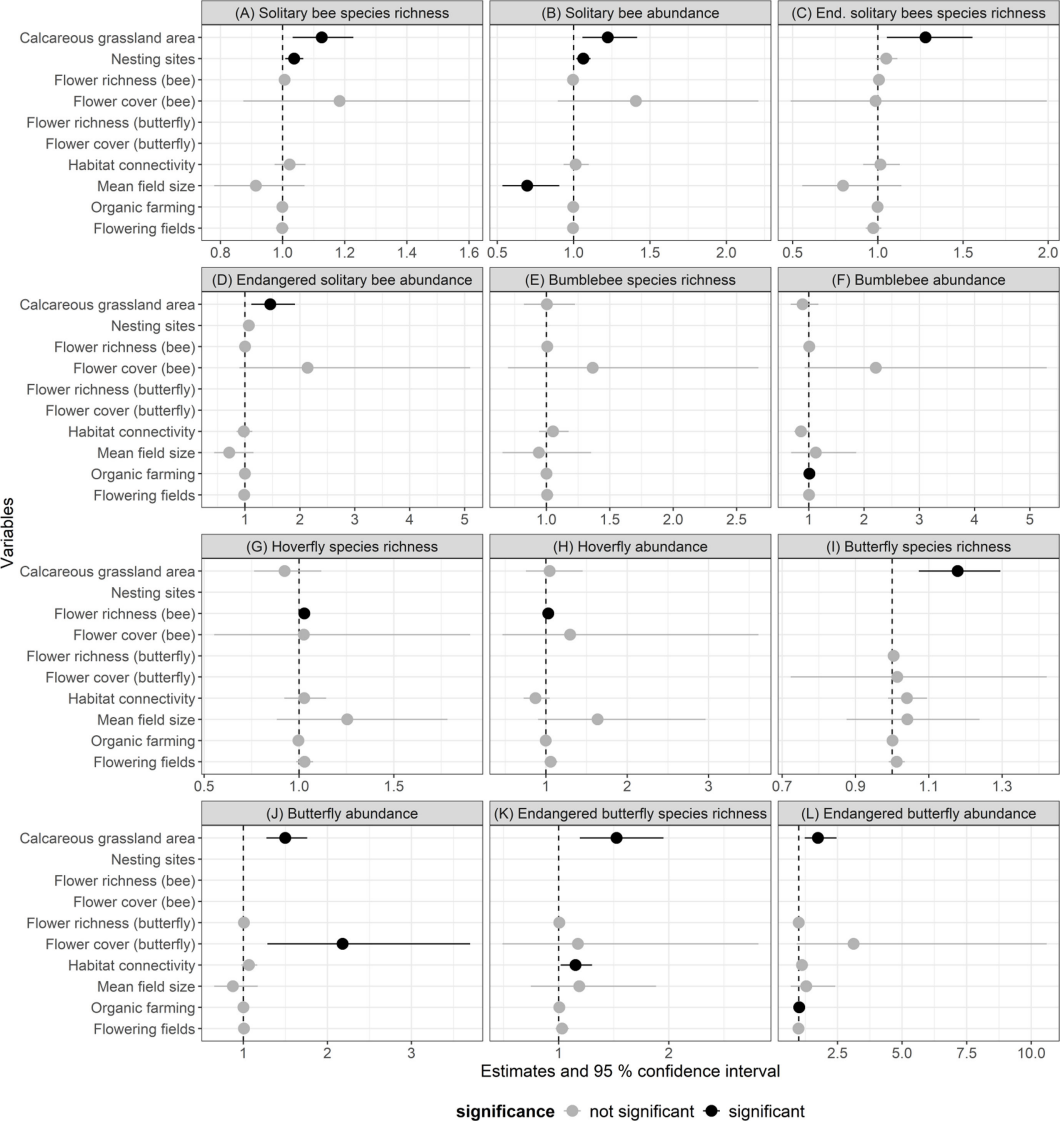
Coefficient estimates and 95% confidence intervals of GLMs analysing the effect of local variables (calcareous grassland area (log10-transformed), flower richness, flower cover (log10-transformed), nesting sites) and landscape variables (habit connectivity, mean field size, organic farming and flowering fields) on pollinator species richness and abundance. Black coefficient estimates and confidence intervals indicate significant results (*p* < 0.05). Parameter estimates have been back-transformed from log-link scale to the response scale, which is why the estimates change around 1 and not 0.

**Figure 4.**
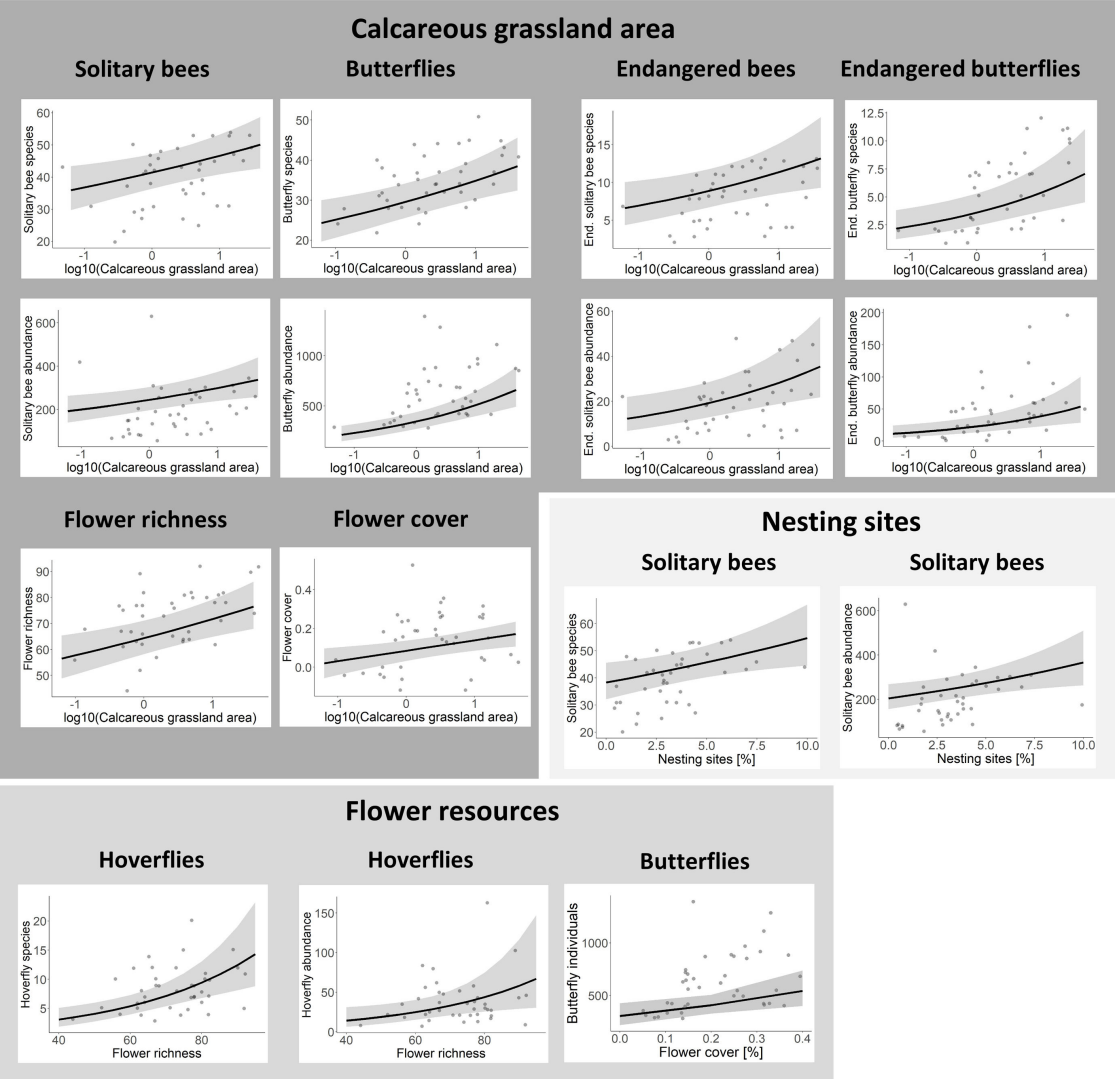
Relationship between (endangered) solitary bee, bumblebee, (endangered) butterfly, hoverfly species richness and abundance and local variables: calcareous grassland area (log10-transformed), flower richness, flower cover and nesting sites. Only significant results of GLMs are shown (*p* < 0.05). A discrepancy between the regression line and the location of the original observed data points (grey points) is possible because the regression line represents the predicted values that have been adjusted by the covariates in the model, with all covariates set to their mean values. Figure with all sub-plots is shown in the supplementary material (electronic supplementary material, Figure S4).

Calcareous grassland area had the strongest effect on solitary bees, butterflies and flower resources. A tenfold increase in calcareous grassland area resulted in an increase in species richness of solitary bees, endangered solitary bees, butterflies and endangered butterflies by 12.6%, 28%, 17.9% and 52.5%, respectively. Furthermore, flower richness and cover of the studied calcareous grasslands increased with increasing area (figures 3 and 4). However, calcareous grassland area had no significant effect on hoverflies and bumblebees (electronic supplementary material, table S1).

Habitat quality affected solitary bees, butterflies and hoverflies. An increasing proportional amount of potential nesting sites had a positive effect on solitary bee species richness and abundance. With additional 5% of the transect area covered with potential nesting sites, 18% more solitary bee species are expected. Endangered solitary bees were not significantly affected by the proportional amount of potential nesting sites (electronic supplementary material, table S1). Hoverfly species richness and abundance increased with increasing flower richness. Flower cover had a significant positive effect on butterfly abundance (figures 3 and 4).

### (b) Landscape effects

**Figure 5.**
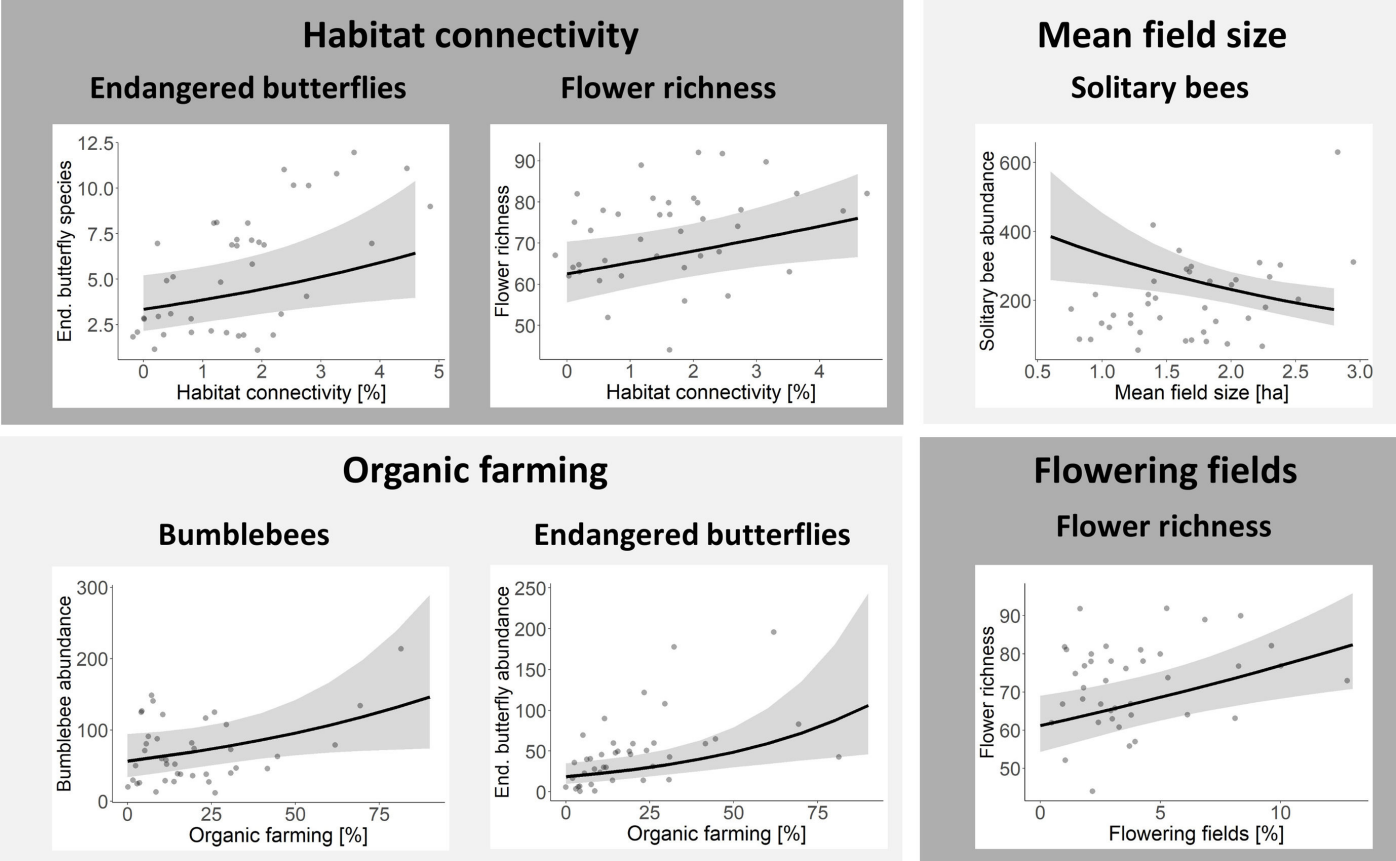
Relationship between (endangered) solitary bee, bumblebee, (endangered) butterfly, hoverfly species richness and abundance and landscape variables: habitat connectivity, mean field size, organic farming and flowering fields. Only significant results of GLMs are shown (*p* < 0.05). A discrepancy between the regression line and the location of the original observed data points (grey points) is possible because the regression line represents the predicted values that have been adjusted by the covariates in the model, with all covariates set to their mean values. Figure with all sub-plots is shown in the supplementary material (electronic supplementary material, figure S4).

Habitat connectivity (i.e. the total amount of calcareous grassland in the surrounding landscape buffer excluding the study site area itself) affected endangered butterfly species and flower resources on calcareous grasslands positively. If habitat connectivity was increased because calcareous grasslands covered an additional 1% of the landscape buffer, endangered butterfly species increased by 15% and flower richness by 4% (figures 3 and 5).

The configuration of the agricultural landscape, as a measure of quality of the agricultural matrix, affected solitary bee abundance: if mean field size of the annual crop fields in the matrix increased by one hectare, solitary bee abundance decreased by 30%. Pollinator species richness and abundance on calcareous grasslands of other pollinator groups were not significantly affected by mean field size. Organic farming (i.e. cover of this AES as a proportion of annual crop fields) had a positive effect on the abundance of bumblebees and endangered butterflies. If an additional 10% of annual crop fields in the landscape buffer were managed organically, bumblebee abundance increased by 10% and endangered butterfly abundance increased by 20%. It should be noted that abundances in this study represent the density of individuals per 1500 and 4500 m² for bees/hoverflies and butterflies, respectively, from which the population size for the total grassland fragment can be derived. Flowering fields (i.e. cover of this AES in proportion to annual crop fields) enhanced flower richness on calcareous grassland. If an additional 1% of annual crop fields in the landscape buffer was converted to flowering fields, 2% more flowering plant species were found on calcareous grassland (figures 3 and 5). Organic farming and flowering fields did not significantly affect pollinator species richness and abundance of other pollinator groups (electronic supplementary material, table S1).

## 4. Discussion

### (a) Local effects

Calcareous grassland area was the strongest predictor of pollinator species richness and thus is in accordance with the species–area relationship predicted by island biogeographic theory [11]. Large calcareous grasslands promote solitary bees and butterflies [64,65] and endangered species even more since these species are often specialized on one habitat [64,66]. At the local scale, larger fragments reduce the risk of extinction and can provide a larger number of food and nesting resources, enabling the coexistence of more pollinator species and larger, more viable populations [12,65,67]. This also becomes clear through the positive relationship between calcareous grassland area and flower resources in this study. In addition to calcareous grassland area, we found that the percentage cover of potential nesting sites was important for solitary bees [17,18]. This is particularly relevant as most bee species in Central Europe are ground nesting [45], and most of the studied calcareous grasslands are located on slopes and have rocky and sandy soil which are suitable nesting substrates [44]. According to our data, already a 5% increase of area providing potential nesting sites increased solitary bee richness by approximately 20%. To achieve the same richness increase based on the species–area relationship a theoretical 30-fold increase of calcareous grassland area would be required, underpinning the value of management options targeted on nesting sites for pollinator conservation. Although calcareous grassland area is the strongest predictor of pollinator species richness and abundance, it is challenging, if not impossible, to expand the size of existing calcareous grasslands. This study indicates that it is not always necessary to increase the calcareous grassland area; rather, improving the quality can be an effective measure. For instance, nesting structures such as open ground and slope edges can be maintained by keeping the area in an open state through mowing, grazing and preventing scrub encroachment.

The positive relationship between flower resources and pollinators on calcareous grasslands also indicates that enhancing the quality of the habitat is a logical course of action when the expansion of calcareous grasslands seems not feasible. Pollinators require nectar and pollen to feed both themselves and their larvae [68,69] and some species are highly restricted in their flower selection [45]. Hence, it is crucial to maintain a high cover of flowers and a diverse range of flowering plant species. This was reflected in the positive relationship between flower cover and butterfly abundance. Recent studies have shown that the availability of flower resources has an impact on the species richness of butterfly [70], solitary bee and bumblebee species [43,71]. However, in our study, solitary bee and butterfly species richness were better explained by calcareous grassland area. In turn, hoverflies benefited from calcareous grasslands through diverse flowering plant species, even when area was held constant. Hoverflies have different requirements than bees and butterflies (i.e. they need a cooler microclimate and their larvae have diverse requirements for their developmental habitat [72]), suggesting that hoverflies do not rely on calcareous grasslands themselves but on flower resources and landscape heterogeneity [47,73]. In general, increasing the size of the habitat is still preferable for pollinator protection. In practice, however, this often encounters difficulties. It is then advisable to pay particular attention to habitat quality, as management changes can significantly impact pollinators. However, smaller calcareous grassland areas are often accompanied by lower habitat quality and loss of ecological processes that lead to a more rapid decay of the ecosystem [12]. Therefore, where it is not feasible to maintain either calcareous grassland area or quality, the focus of conservation approaches should be beyond local habitats at landscape scales.

### (b) Landscape effects

Habitat connectivity—measured as the amount of calcareous grasslands at the landscape scale—positively influenced endangered butterfly species. This positive pattern has also been found in other semi-natural grasslands [19,74] and can be explained by increased dispersal of butterflies. Higher colonization rates reduce the risk of extinction in fragmented habitats [75]. The connectivity effects indicate the importance of immigration for the survival of endangered species. A positive effect of habitat connectivity on flower richness suggests that habitat connectivity enhances seed dispersal and genetic diversity of plants [76]. This, in turn, can promote pollinators in their local habitats by diversifying their food resources.

Habitat connectivity may also be important for bees, as the presence of multiple habitat fragments is necessary for their survival as suggested by the metapopulation theory [77]. However, in our study region, connectivity was not a limiting factor for solitary bees. Landscape variables regarding agricultural management were more important. Landscapes with smaller crop fields were beneficial for solitary bee abundance, and our data suggest that a reduction of field sizes is an effective measure to promote solitary bees where calcareous grassland area cannot be enlarged. To increase the abundance of solitary bees by 20%, a reduction of the average field size by 0.5 ha is already sufficient, while the calcareous grassland area would have to be six times larger than before to achieve the same effect. It should be noted that the density of bee individuals per transect (1500 m²) is being discussed here, rather than the total abundance of the calcareous grassland fragment. An increase in calcareous grassland area not only leads to an increase in densities per transect, but also to an increase in population sizes due to the effect of the increase in area alone. The effect achieved by increasing calcareous grassland area is therefore many times greater and should therefore be favoured in nature conservation measures. However, smaller fields result in longer and more edge structures between adjacent annual crop fields [27]. Structures such as field margins, hedgerows and small open paths provide additional food and nesting resources by having a higher flower cover and experiencing fewer disturbances. Additionally, field edges receive less management intensity than field centres and are therefore better habitats for pollinators. The additional colonization of plants in the field edges has the effect of an increased food supply. Pollinators also colonize the less intensively managed field edges [26,78]. Field boundaries and edges therefore enhance the presence of food and nesting sites and connectivity since pollinators can more easily move across crop fields [79,80]. This study emphasizes that dividing large crop fields into smaller ones may be an effective approach to benefit solitary bees not only inside the agricultural matrix but even in high-value habitats. This relatively easy-to-implement measure could be accompanied by an establishment of new hedgerows and unmanaged field margins to further increase structural heterogeneity at the landscape scale. Moreover, the study indicates that adaptations in landscape management can have a more pronounced impact than changes in conventional landscape variables, such as calcareous grassland area and connectivity, which are frequently challenging to implement in practice. However, mean field size did not affect bumblebees, hoverflies or butterflies, despite the likelihood of a more structurally diverse agriculture and increasing food and shelter resources resulting from it [81–83].

For bumblebees and endangered butterflies on calcareous grasslands, organic farming in the surrounding landscape was found to be a determining factor, likely due to the positive aspects of organic compared with conventional farming. Pollinators are less exposed to insecticides in calcareous grassland adjacent to organic fields [49,84], and reduced herbicide use leads to a higher flower cover of non-crop vegetation in organically managed fields [29]. Bumblebees and endangered butterflies likely benefited from higher floral and larvae plant resources [23,85]. In general, few studies have tested the effect of organic farming on pollinator species richness and abundance at the landscape scale [30]. It is therefore all the more remarkable that this study showed positive effects of organic farming on bumblebees and butterflies in protected high-value habitats. This study also shows that increasing the cover of organic farming can be an effective measure in pollinator conservation where the expansion of calcareous grassland area is not feasible. Our data suggest that there is a 20% increase in endangered butterfly abundance if an additional 10% of the surrounding crop cover is converted to organic farming. To achieve the same result, the calcareous grassland area would have to be 2.25-times larger. As previously stated, these are densities per transect and not total abundances of the grassland fragment. An increase in calcareous grassland area is also associated with an increase in population sizes and, therefore, should be favoured as a measure. For bumblebee abundances, organic farming was the only influencing variable and resulted in a 10% increase in abundances per additional 10% of annual crop cover managed organically. Solitary bee abundance was not affected by the cover of organic farming but by mean field size of annual crops in the surrounding landscape, suggesting that for solitary bees in calcareous grasslands, the structure of the agricultural landscape is more important than management type. However, Holzschuh *et al*. [30] showed that a high organic land cover in the landscape had a positive impact on wild bee species richness and density in fallow strips due to increased flower resources. While other studies show that AES are not effective for conserving endangered species in crop fields themselves [84,86], our study suggests that organic farming can support endangered butterflies as well as bumblebees on landscape scales and in high-value pollinator habitats.

We found no direct effect of flowering fields in the surrounding landscape on pollinator richness or abundance on calcareous grasslands. Recent studies provide evidence for the effectiveness of flowering fields along crop field edges for pollinator species richness and abundance in field edges [31,87]. It becomes clear that flowering fields attract many pollinators and are advantageous compared with other habitats, such as flowerless crop fields. The low gradient of flowering fields in the landscape in our study could explain missing correlations. However, we found a positive effect of flowering fields on flower richness in calcareous grasslands, presumably through seed dispersal, underpinning the importance of using autochthonous origins for seed mixtures. In addition, the seed mixtures eligible for federal funding can contain over 40 plant species, including those found on calcareous grasslands. Therefore, pollinators profit at least indirectly from sown flowering fields in the agricultural landscape. Hoverflies were not influenced by any landscape variable. Since hoverflies are not specialised on calcareous grasslands, heterogeneous landscapes, in general may be more beneficial [73].

## 5. Conclusion

Our study reveals the potential of combined management options focused on improving local habitat quality and the creation of beneficial AES in the surrounding landscape as an alternative to the classically considered variables of calcareous grassland area and connectivity to ensure the long-term survival of diverse and partially endangered pollinator groups. Future conservation approaches should focus on the preservation of calcareous grassland fragments with attention to area and quality to counteract pollinator species decline through habitat loss and ecosystem decay. In practice, however, it is often not possible to increase the calcareous grassland area or connectivity in order to improve pollinator richness. This study shows that adapted landscape management and an improvement of habitat quality, especially of nesting sites, can be an effective and more feasible method for pollinator conservation. The configuration of agriculture and AES in the surrounding landscape favours pollinators not only within the agricultural matrix but even in embedded high-value habitats. Small fields and organically managed crops support the conservation of different wild pollinator groups, including endangered species, in protected high-value habitats like calcareous grasslands by providing additional flower and nesting resources at the landscape scale. Nonetheless, more efforts to expand the area and connectivity of high-value habitats will be necessary to mitigate extinction debts in fragmented habitats [67] and ensure the long-term preservation of pollinator richness in human-modified landscapes.

## Data Availability

All datasets and the R code supporting this article have been made publicly available at the Dryad Digital Repository [88]. Supplementary material is available online [89].

## References

[1] Uhler J *et al*. 2021 Relationship of insect biomass and richness with land use along a climate gradient. Nat. Commun. **12**, 5946. (10.1038/s41467-021-26181-3) 34642336 PMC8511018

[2] Wagner DL, Grames EM, Forister ML, Berenbaum MR, Stopak D. 2021 Insect decline in the Anthropocene: death by a thousand cuts. Proc. Natl Acad. Sci. USA **118**, e2023989118. (10.1073/pnas.2023989118) 33431573 PMC7812858

[3] Seibold S *et al*. 2019 Arthropod decline in grasslands and forests is associated with landscape-level drivers. Nature **574**, 671–674. (10.1038/s41586-019-1684-3) 31666721

[4] Klein AM, Vaissière BE, Cane JH, Steffan-Dewenter I, Cunningham SA, Kremen C, Tscharntke T. 2007 Importance of pollinators in changing landscapes for world crops. Proc. Biol. Sci. **274**, 303–313. (10.1098/rspb.2006.3721) 17164193 PMC1702377

[5] Ollerton J, Winfree R, Tarrant S. 2011 How many flowering plants are pollinated by animals? Oikos **120**, 321–326. (10.1111/j.1600-0706.2010.18644.x)

[6] Hendricks F *et al*. 2007 How landscape structure, land‐use intensity and habitat diversity affect components of total arthropod diversity in agricultural landscapes. J. Appl. Ecol. **44**, 340–351. (10.1111/j.1365-2664.2006.01270.x)

[7] Öckinger E, Smith HG. 2007 Semi‐natural grasslands as population sources for pollinating insects in agricultural landscapes. J. Appl. Ecol. **44**, 50–59. (10.1111/j.1365-2664.2006.01250.x)

[8] Boetzl FA *et al*. 2021 A multitaxa assessment of the effectiveness of agri-environmental schemes for biodiversity management. Proc. Natl Acad. Sci. USA **118**, e2016038118. (10.1073/pnas.2016038118) 33649216 PMC7958248

[9] van Swaay CAM. 2002 The importance of calcareous grasslands for butterflies in Europe. Biol. Conserv. **104**, 315–318. (10.1016/s0006-3207(01)00196-3)

[10] Poschlod P, WallisDeVries MF. 2002 The historical and socioeconomic perspective of calcareous grasslands—lessons from the distant and recent past. Biol. Conserv. **104**, 361–376. (10.1016/s0006-3207(01)00201-4)

[11] MacArthur RH, Wilson EO. 1963 An equilibrium theory of insular zoogeography. Evolution **17**, 373–387. (10.1111/j.1558-5646.1963.tb03295.x)

[12] Chase JM, Blowes SA, Knight TM, Gerstner K, May F. 2020 Ecosystem decay exacerbates biodiversity loss with habitat loss. Nature **584**, 238–243. (10.1038/s41586-020-2531-2) 32728213

[13] Krauss J, Alfert T, Steffan‐Dewenter I. 2009 Habitat area but not habitat age determines wild bee richness in limestone quarries. J. Appl. Ecol. **46**, 194–202. (10.1111/j.1365-2664.2008.01582.x)

[14] Steffan‐Dewenter I. 2003 Importance of habitat area and landscape context for species richness of bees and wasps in fragmented orchard meadows. Conserv. Biol. **17**, 1036–1044. (10.1046/j.1523-1739.2003.01575.x)

[15] Fantinato E, Sonkoly J, Török P, Buffa G. 2021 Patterns of pollination interactions at the community level are related to the type and quantity of floral resources. Funct. Ecol. **35**, 2461–2471. (10.1111/1365-2435.13915)

[16] Roulston TH, Goodell K. 2011 The role of resources and risks in regulating wild bee populations. Annu. Rev. Entomol. **56**, 293–312. (10.1146/annurev-ento-120709-144802) 20822447

[17] Hopfenmüller S, Steffan-Dewenter I, Holzschuh A. 2014 Trait-specific responses of wild bee communities to landscape composition, configuration and local factors. PLoS One **9**, e104439. (10.1371/journal.pone.0104439) 25137311 PMC4138035

[18] Potts SG, Vulliamy B, Roberts S, O’Toole C, Dafni A, Ne’eman G, Willmer P. 2005 Role of nesting resources in organising diverse bee communities in a Mediterranean landscape. Ecol. Entomol. **30**, 78–85. (10.1111/j.0307-6946.2005.00662.x)

[19] Brückmann SV, Krauss J, Steffan‐Dewenter I. 2010 Butterfly and plant specialists suffer from reduced connectivity in fragmented landscapes. J. Appl. Ecol. **47**, 799–809. (10.1111/j.1365-2664.2010.01828.x)

[20] Bergman KO, Dániel-Ferreira J, Milberg P, Öckinger E, Westerberg L. 2018 Butterflies in Swedish grasslands benefit from forest and respond to landscape composition at different spatial scales. Landsc. Ecol **33**, 2189–2204. (10.1007/s10980-018-0732-y)

[21] Vandermeer J, Carvajal R. 2001 Metapopulation dynamics and the quality of the matrix. Am. Nat. **158**, 211–220. (10.1086/321318) 18707319

[22] Cook WM, Lane KT, Foster BL, Holt RD. 2002 Island theory, matrix effects and species richness patterns in habitat fragments. Ecol. Lett. **5**, 619–623. (10.1046/j.1461-0248.2002.00366.x)

[23] Carrié R, Ekroos J, Smith HG. 2018 Organic farming supports spatiotemporal stability in species richness of bumblebees and butterflies. Biol. Conserv. **227**, 48–55. (10.1016/j.biocon.2018.08.022)

[24] Holzschuh A, Dormann CF, Tscharntke T, Steffan-Dewenter I. 2013 Mass-flowering crops enhance wild bee abundance. Oecologia **172**, 477–484. (10.1007/s00442-012-2515-5) 23114428 PMC3655217

[25] Holzschuh A, Steffan‐dewenter I, Kleijn D, Tscharntke T. 2007 Diversity of flower‐visiting bees in cereal fields: effects of farming system, landscape composition and regional context. J. Appl. Ecol. **44**, 41–49. (10.1111/j.1365-2664.2006.01259.x)

[26] Clough Y, Holzschuh A, Gabriel D, Purtauf T, Kleijn D, Kruess A, Steffan‐dewenter I, Tscharntke T. 2007 Alpha and beta diversity of arthropods and plants in organically and conventionally managed wheat fields. J. Appl. Ecol. **44**, 804–812. (10.1111/j.1365-2664.2007.01294.x)

[27] Tscharntke T, Grass I, Wanger TC, Westphal C, Batáry P. 2021 Beyond organic farming: harnessing biodiversity-friendly landscapes. Trends Ecol. Evol. **36**, 919–930. (10.1016/j.tree.2021.06.010) 34362590

[28] Clough Y, Kirchweger S, Kantelhardt J. 2020 Field sizes and the future of farmland biodiversity in European landscapes. Conserv. Lett. **13**, e12752. (10.1111/conl.12752) 33519969 PMC7816254

[29] Geppert C, Hass A, Földesi R, Donkó B, Akter A, Tscharntke T, Batáry P. 2020 Agri‐environment schemes enhance pollinator richness and abundance but bumblebee reproduction depends on field size. J. Appl. Ecol. **57**, 1818–1828. (10.1111/1365-2664.13682)

[30] Holzschuh A, Steffan‐Dewenter I, Tscharntke T. 2008 Agricultural landscapes with organic crops support higher pollinator diversity. Oikos **117**, 354–361. (10.1111/j.2007.0030-1299.16303.x)

[31] Lowe EB, Groves R, Gratton C. 2021 Impacts of field-edge flower plantings on pollinator conservation and ecosystem service delivery – A meta-analysis. Agric. Ecosyst. Environ. **310**, 107290. (10.1016/j.agee.2020.107290)

[32] Holzschuh A, Steffan‐Dewenter I, Tscharntke T. 2010 How do landscape composition and configuration, organic farming and fallow strips affect the diversity of bees, wasps and their parasitoids? J. Anim. Ecol. **79**, 491–500. (10.1111/j.1365-2656.2009.01642.x) 20015213

[33] Rundlöf M, Bengtsson J, Smith HG. 2008 Local and landscape effects of organic farming on butterfly species richness and abundance. J. Appl. Ecol. **45**, 813–820. (10.1111/j.1365-2664.2007.01448.x)

[34] DWD Climate Data Center (CDC). 2023 Jahresmittel der Stationsmessungen der Lufttemperatur in 2 m Höhe in °C für Deutschland. See https://cdc.dwd.de/portal/202209231028/mapview (accessed 24 October 2023).

[35] DWD Climate Data Center (CDC). 2023 Jahressumme der Stationsmessungen der Niederschlagshöhe in mm für Deutschland. See https://cdc.dwd.de/portal/202209231028/mapview (accessed 24 October 2023).

[36] Google LLC. 2023 Google Earth v 10.71.0.2. See https://earth.google.com/web/@34.27381363,-173.76670724,-40931.16409662a,22292683.93853188d,35y,0h,0t,0r/data=CgRCAggBQgIIAEoNCP___________wEQAA.

[37] Doppler G, Fiebig M, Freudenberger W, Glaser S, Meyer R, Pürner T, Rohrmüller J, Schwerd K. 2004 GeoBavaria 600 millionen jahre bayern. Munich, Germany: Bayerisches Geologisches Landesamt.

[38] Boehmer HJ. 1994 Die Halbtrockenrasen der Fränkischen Alb. Mitteilungen Der Fränkischen Geographischen Gesellschaft **41**, 323–344.

[39] Voith J, Doczkal D, Dubitzky A, Hopfenmüller S, Mandery K, Scheuchl E, Schuberth J, Weber K. 2021 Rote Liste und Gesamtartenliste Bayern–Bienen–Hymenoptera, Anthophila. Augsburg, Germany: Bayerisches Landesamt für Umwelt.

[40] Connor EF, Courtney AC, Yoder JM. 2000 Individuals–area relationships: the relationship between animal population density and area. Ecology **81**, 734–748. (10.1890/0012-9658(2000)081[0734:iartrb]2.0.co;2)

[41] Watling JI *et al*. 2020 Support for the habitat amount hypothesis from a global synthesis of species density studies. Ecol. Lett. **23**, 674–681. (10.1111/ele.13471) 32043741

[42] Esri, M. 2022 World imagery. Earthstar Geographics & GIS User Community. See https://www.arcgis.com/home/item.html?id=10df2279f9684e4a9f6a7f08febac2a9.

[43] Gardein H, Fabian Y, Westphal C, Tscharntke T, Hass A. 2022 Ground-nesting bees prefer bare ground areas on calcareous grasslands. Glob. Ecol. Conserv. **39**, e02289. (10.1016/j.gecco.2022.e02289)

[44] Sardiñas HS, Kremen C. 2014 Evaluating nesting microhabitat for ground-nesting bees using emergence traps. Basic Appl. Ecol. **15**, 161–168. (10.1016/j.baae.2014.02.004)

[45] Westrich P. 1996 Habitat requirements of central european bees and the problems of partial habitats. London, UK: The Linnean Society of London and The International Bee Research Association.

[46] Zurbuchen A, Landert L, Klaiber J, Müller A, Hein S, Dorn S. 2010 Maximum foraging ranges in solitary bees: only few individuals have the capability to cover long foraging distances. Biol. Conserv. **143**, 669–676. (10.1016/j.biocon.2009.12.003)

[47] Meyer B, Jauker F, Steffan-Dewenter I. 2009 Contrasting resource-dependent responses of hoverfly richness and density to landscape structure. Basic Appl. Ecol. **10**, 178–186. (10.1016/j.baae.2008.01.001)

[48] Osborne JL, Martin AP, Carreck NL, Swain JL, Knight ME, Goulson D, Hale RJ, Sanderson RA. 2008 Bumblebee flight distances in relation to the forage landscape. J. Anim. Ecol. **77**, 406–415. (10.1111/j.1365-2656.2007.01333.x) 17986207

[49] Federal Ministry of Food and Agriculture (BMEL). 2022 Organic farming in Germany. See https://www.bmel.de/SharedDocs/Downloads/EN/Publications/Organic-Farming-in-Germany.pdf?__blob=publicationFile&v=4.

[50] Esri Inc. 2018 ArcGIS Pro 2.2.0. See https://www.esri.com/de-de/arcgis/products/arcgis-pro/overview.

[51] Arif S, MacNeil MA. 2023 Applying the structural causal model framework for observational causal inference in ecology. Ecol. Monogr. **93**. (10.1002/ecm.1554)

[52] R Development Core Team. 2023 R v 4.3.1 and RStudio v 2023.06.1. See https://cran.r-project.org/bin/windows/base/old/4.3.1/ and https://dailies.rstudio.com/version/2023.06.1+524/.

[53] Wickham H. 2016 tidyverse: easily install and load the ‘Tidyverse’. See https://cran.r-project.org/web/packages/tidyverse/index.html.

[54] Venables WN, Ripley BD. 2002 Modern applied statistics with s, 4th edition. New York, NY: Springer.

[55] Textor J, Zander B, Ankan A. 2023 Graphical analysis of structural causal models. Version 0.3-4. See https://cran.r-project.org/web/packages/dagitty/dagitty.pdf.

[56] Hartig F, Lohse L. 2022 DHARMa: residual diagnostics for hierarchical (multi-level/mixed) regression models. Version 0.4.6. See https://cran.r-project.org/web/packages/DHARMa/index.html.

[57] Fox J, Weisberg S, Price B. 2019 car: companion to applied regression. Version 3.1-3. See https://cran.r-project.org/web/packages/car/index.html.

[58] Bjornstad ON. 2008 ncf: dsatial covariance functions. (10.32614/cran.package.ncf)

[59] Lüdecke D *et al*. 2024 Package ‘performance’: assessment of regression models performance. Version 0.11.0. See https://cran.r-project.org/web/packages/performance/performance.pdf.

[60] Arel-Bundock V, Augusto Diniz M, Greifer N, Bacher E. 2024 Package ‘marginaleffects’: means, and hypothesis tests. Version 0.20.1. See https://cran.r-project.org/web/packages/marginaleffects/marginaleffects.pdf.

[61] Arel-Bundock V, Gassen J, Eastwood N, Huntington-Klein N, Schwarz M, Elbers B, McDermott G, Wallrich L. 2019 Package ‘modelsummary’: summary tables and plots for statistical models and data: beautiful, customizable, and publication-ready. Version 2.1.0. See https://cran.r-project.org/web/packages/modelsummary/modelsummary.pdf (accessed 12 March 2024).

[62] Wickham H. 2007 ggplot2: create elegant data visualisations using the grammar of graphics. Version 3.5.1. See https://cran.r project.org/web/packages/ggplot2/index.html.

[63] Lüdecke D, Aust F, Crawley S, Ben-Shachar MS, Anderson SC. 2017 ggeffects: create tidy data frames of marginal effects for ‘ggplot’ from model outputs. Version 1.6.0. See https://cran.r-project.org/web/packages/ggeffects/ggeffects.pdf.

[64] Botham MS, Fernandez-Ploquin EC, Brereton T, Harrower CA, Roy DB, Heard MS. 2015 Lepidoptera communities across an agricultural gradient: how important are habitat area and habitat diversity in supporting high diversity? J. Insect Conserv. **19**, 403–420. (10.1007/s10841-015-9760-y)

[65] Jauker B, Krauss J, Jauker F, Steffan-Dewenter I. 2013 Linking life history traits to pollinator loss in fragmented calcareous grasslands. Landsc. Ecol. **28**, 107–120. (10.1007/s10980-012-9820-6)

[66] Krauss J, Steffan-Dewenter I, Tscharntke T. 2003 Local species immigration, extinction, and turnover of butterflies in relation to habitat area and habitat isolation. Oecologia **137**, 591–602. (10.1007/s00442-003-1353-x) 14505022

[67] Kuussaari M *et al*. 2009 Extinction debt: a challenge for biodiversity conservation. Trends Ecol. Evol. **24**, 564–571. (10.1016/j.tree.2009.04.011) 19665254

[68] Curtis RJ, Brereton TM, Dennis RLH, Carbone C, Isaac NJB. 2015 Butterfly abundance is determined by food availability and is mediated by species traits. J. Appl. Ecol. **52**, 1676–1684. (10.1111/1365-2664.12523)

[69] Müller A, Diener S, Schnyder S, Stutz K, Sedivy C, Dorn S. 2006 Quantitative pollen requirements of solitary bees: Implications for bee conservation and the evolution of bee–flower relationships. Biol. Conserv. **130**, 604–615. (10.1016/j.biocon.2006.01.023)

[70] Luppi M, Dondina O, Orioli V, Bani L. 2018 Local and landscape drivers of butterfly richness and abundance in a human-dominated area. Agric. Ecosyst. Environ. **254**, 138–148. (10.1016/j.agee.2017.11.020)

[71] Neumüller U, Burger H, Krausch S, Blüthgen N, Ayasse M. 2020 Interactions of local habitat type, landscape composition and flower availability moderate wild bee communities. Landsc. Ecol. **35**, 2209–2224. (10.1007/s10980-020-01096-4)

[72] Hoiß B. 2020 Schwebfliegen – vergessene Helfer mit faszinierender Ökologie. ANLiegen Nat **42**, 81–90. https://www.anl.bayern.de/publikationen/anliegen/doc/an42106hoiss_2020_schwebfliegen.pdf

[73] Schirmel J, Albrecht M, Bauer P, Sutter L, Pfister SC, Entling MH. 2018 Landscape complexity promotes hoverflies across different types of semi‐natural habitats in farmland. J. Appl. Ecol. **55**, 1747–1758. (10.1111/1365-2664.13095)

[74] Öckinger E, Smith HG. 2006 Landscape composition and habitat area affects butterfly species richness in semi-natural grasslands. Oecologia **149**, 526–534. (10.1007/s00442-006-0464-6) 16775707

[75] Fernández‐Chacón A, Stefanescu C, Genovart M, Nichols JD, Hines JE, Páramo F, Turco M, Oro D. 2014 Determinants of extinction‐colonization dynamics in Mediterranean butterflies: the role of landscape, climate and local habitat features. J. Anim. Ecol. **83**, 276–285. (10.1111/1365-2656.12118) 23957287

[76] Clough Y *et al*. 2014 Density of insect‐pollinated grassland plants decreases with increasing surrounding land‐use intensity. Ecol. Lett. **17**, 1168–1177. (10.1111/ele.12325) 25040328

[77] Hanski I, Ovaskainen O. 2003 Metapopulation theory for fragmented landscapes. Theor. Popul. Biol. **64**, 119–127. (10.1016/s0040-5809(03)00022-4) 12804876

[78] Krimmer E, Martin EA, Krauss J, Holzschuh A, Steffan-Dewenter I. 2019 Size, age and surrounding semi-natural habitats modulate the effectiveness of flower-rich agri-environment schemes to promote pollinator visitation in crop fields. Agric. Ecosyst. Environ. **284**, 106590. (10.1016/j.agee.2019.106590)

[79] Hass AL *et al*. 2018 Landscape configurational heterogeneity by small-scale agriculture, not crop diversity, maintains pollinators and plant reproduction in western Europe. Proc. Biol. Sci. **285**. (10.1098/rspb.2017.2242) PMC5829195 29445017

[80] Morandin LA, Kremen C. 2013 Hedgerow restoration promotes pollinator populations and exports native bees to adjacent fields. Ecol. Appl. **23**, 829–839. (10.1890/12-1051.1) 23865233

[81] Muljar R, Viik E, Marja R, Svilponis E, Jogar K, Karise R, Mänd M. 2010 The effect of field size on the number of bumble bees. Agron. Res. **2**, 357–360.

[82] Pywell RF, Warman EA, Sparks TH, Greatorex-Davies JN, Walker KJ, Meek WR, Carvell C, Petit S, Firbank LG. 2004 Assessing habitat quality for butterflies on intensively managed arable farmland. Biol. Conserv. **118**, 313–325. (10.1016/j.biocon.2003.09.011)

[83] Sutherland J, Sullivan M, Poppy G. 2001 Distribution and abundance of aphidophagous hoverflies (Diptera: Syrphidae) in wildflower patches and field margin habitats. Agri For. Entomol. **3**, 57–64. (10.1046/j.1461-9563.2001.00090.x)

[84] Kleijn D *et al*. 2006 Mixed biodiversity benefits of agri‐environment schemes in five European countries. Ecol. Lett. **9**, 243–254. (10.1111/j.1461-0248.2005.00869.x) 16958888

[85] Goded S, Ekroos J, Azcárate JG, Guitián JA, Smith HG. 2019 Effects of organic farming on plant and butterfly functional diversity in mosaic landscapes. Agric. Ecosyst. Environ. **284**, 106600. (10.1016/j.agee.2019.106600)

[86] Scheper J, Holzschuh A, Kuussaari M, Potts SG, Rundlöf M, Smith HG, Kleijn D. 2013 Environmental factors driving the effectiveness of European agri‐environmental measures in mitigating pollinator loss—a meta‐analysis. Ecol. Lett. **16**, 912–920. (10.1111/ele.12128) 23714393

[87] Ganser D, Albrecht M, Knop E. 2021 Wildflower strips enhance wild bee reproductive success. J. Appl. Ecol. **58**, 486–495. (10.1111/1365-2664.13778)

[88] Biegerl C, Holzschuh A, Tanner B, Sponsler D, Krauss J, Zhang J, Steffan-Dewenter I. 2025 Landscape management can foster pollinator richness in fragmented high-value habitats. Dryad Digital Repository. (10.5061/dryad.dncjsxm90)39904381

[89] Biegerl C, Holzschuh A, Tanner B, Sponsler D, Krauss J, Zhang J. 2025 Supplementary material from: Landscape management can foster pollinator richness in fragmented high-value habitats. Figshare. (10.6084/m9.figshare.c.7634285)39904381

